# Supplementary material for: Dose-sparing effect of lapatinib co-administered with a high-fat enteral nutrition emulsion: preclinical pharmacokinetic study
Source: PeerJ. 2023 Oct 9;11:e16207. doi: 10.7717/peerj.16207 (PMC10569162; doi:10.7717/peerj.16207)
Supplement: Supplemental Information 2 [file peerj-11-16207-s002.doc]

# Dose-sparing effect of lapatinib co-administered with a high-fat enteral nutrition emulsion: preclinical pharmacokinetic study

Junfeng Zhu^1,2^, Gaoqi Xu^1,2^, Dihong Yang^1,2^, Yu Song^1,2^, Yinghui Tong^1,2^, Sisi Kong^1,2^, Haiying Ding^1,2^, Luo Fang^1,2,*^

^1^ Department of Pharmacy, Zhejiang Cancer Hospital, Hangzhou 310022, China

^2^ Hangzhou Institute of Medicine (HIM), Chinese Academy of Sciences, Hangzhou 310018, China

^*^Correspondence: Luo Fang, Department of Pharmacy, Zhejiang Cancer Hospital, Hangzhou 310022, China. Email: fangluo@zjcc.org.cn

**Supporting Information**

**Table S1** Nutrient formulation of TPF-T

**Table S2** Intra- and inter-day precision and accuracy of lapatinib in rabbit plasma

**Table S3** Extraction recovery and matrix effect of lapatinib in rabbit plasma

**Table S4** Stability (%) of lapatinib in rabbit plasma under different conditions

**Figure S1** Calibration curve of lapatinib in rabbit plasma

**Table S1** Nutrient formulation of TPF-T

| Nutrient | Per 200ml |
| --- | --- |
| Calories (kcal) | 260 |
| Protein (g) | 11.7 |
| Fat (g) | 14.4 |
| Carbohydrate (g) | 20.8 |
| Fiber (g) | 2.6 |
| Sodium (mg) | 160 |
| Potassium (mg) | 344 |
| Chlorine (mg) | 248 |
| Calcium (mg) | 100 |
| Phosphorus (mg) | 100 |
| Magnesium (mg) | 44 |
| Iron (mg) | 2.6 |
| Zinc (mg) | 2 |
| Copper (mg) | 0.26 |
| Manganese (mg) | 0.54 |
| Iodine (μg) | 26.6 |
| Fluorine (mg) | 0.26 |
| Chromium (μg) | 13.2 |
| Molybdenum (μg) | 20 |
| Selenium (μg) | 13.4 |
| Vitamin A (mg) | 0.4 |
| Vitamin D3 (μg) | 0.92 |
| Vitamin E (mg) | 5.4 |
| Vitamin K1 (μg) | 13.2 |
| Vitamin B1 (mg) | 0.26 |
| Vitamin B2 (mg) | 0.34 |
| Niacinamide (mg) | 2.4 |
| Vitamin B6 (mg) | 0.32 |
| Vitamin B12 (μg) | 0.52 |
| Pantothenic acid (mg) | 0.92 |
| Biotin (μg) | 26 |
| Folic acid (μg) | 26 |
| Vitamin C (mg) | 16 |
| Choline (μg) | 53.2 |

**Table S2** Intra- and inter-day precision and accuracy of lapatinib in rabbit plasma

| Nominal concentration  (ng/mL) | Intra-day (n = 5) | | | Inter-day (n = 15) | | |
| --- | --- | --- | --- | --- | --- | --- |
|  | Mean ± SD  (ng/mL) | Accuracy  (RE, %) | Precision  (RSD, %) | Mean ± SD  (ng/mL) | Accuracy  (RE, %) | Precision  (RSD, %) |
| 5 | 5.27 ± 0.42 | 5.46 | 7.90 | 5.05 ± 0.40 | 0.92 | 7.98 |
| 10 | 10.03 ± 0.21 | 0.27 | 2.04 | 10.76 ± 0.64 | 7.56 | 5.95 |
| 500 | 482.53 ± 4.22 | –3.49 | 0.87 | 521.14 ± 29.63 | 4.23 | 5.68 |
| 800 | 783.22 ± 6.98 | –2.10 | 0.89 | 807.62 ± 24.12 | 0.95 | 2.99 |

**Table S3** Extraction recovery and matrix effect of lapatinib in rabbit plasma

| Nominal concentration  (ng/mL) | Extraction recovery (n = 5) | | Matrix effect (n = 6) | |
| --- | --- | --- | --- | --- |
|  | Mean ± SD (%) | RSD (%) | Mean ± SD (%) | RSD (%) |
| 10 | 99.46 ± 1.93 | 1.94 | 88.32 ± 3.01 | 3.45 |
| 500 | 100.11 ± 2.90 | 2.90 | NE | NE |
| 800 | 103.40 ± 2.09 | 2.02 | 88.70 ± 1.03 | 1.16 |

**Abbreviation:** NE, not evaluated.

**Table S4** Stability (%) of lapatinib in rabbit plasma under different conditions

| Nominal concentration  (ng/mL) | Short-Term^a^ | Long-Term^b^ | Freeze–thaw^c^ | Post–Preparation^d^ | Autosampler^e^ |
| --- | --- | --- | --- | --- | --- |
| 10 | 124.03 ± 4.22 | 97.20 ± 4.69 | 100.37 ± 4.14 | 113.77 ± 3.65 | 108.86 ± 1.42 |
| 800 | 101.16 ± 0.42 | 102.05 ± 0.57 | 102.01 ± 1.86 | 101.86 ± 1.06 | 101.98 ± 0.46 |

**Notes:** ^a^Ice bath for 3 h; ^b^–80 °C refrigerator for 14 days; ^c^three freeze–thaw cycles; ^d^ice bath for 3 h after sample preparation; ^e^autosampler room (10 °C) for 4 h after sample preparation.


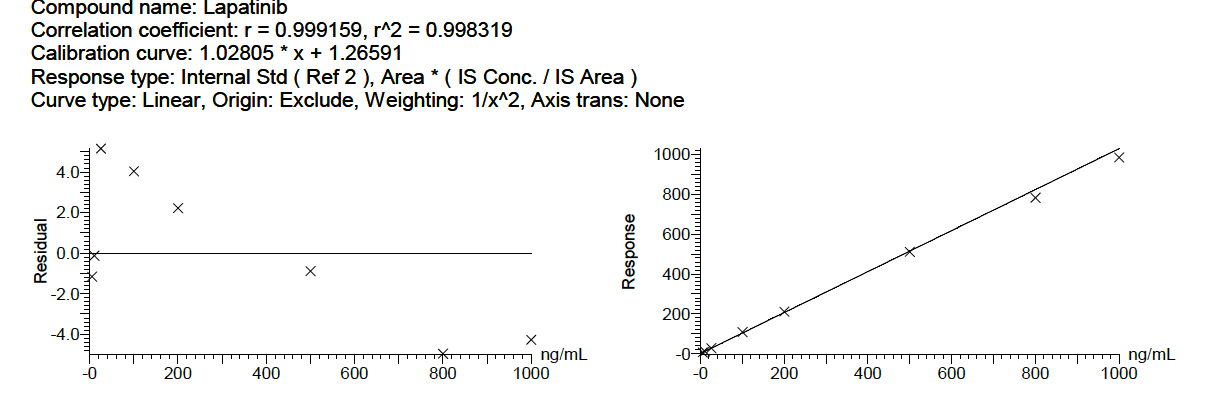


**Figure S1** Calibration curve of lapatinib in rabbit plasma
